# A model predictive control approach to optimally devise a two‐dose vaccination rollout: A case study on COVID‐19 in Italy

**DOI:** 10.1002/rnc.5728

**Published:** 2021-08-25

**Authors:** Francesco Parino, Lorenzo Zino, Giuseppe C. Calafiore, Alessandro Rizzo

**Affiliations:** ^1^ Department of Electronics and Telecommunications Politencico di Torino Turin Italy; ^2^ Faculty of Science and Engineering University of Groningen Groningen the Netherlands; ^3^ Institute of Electronics, Information Engineering and Telecommunications (IEIIT) Consiglio Nazionale delle Ricerche Turin Italy; ^4^ Office of Innovation New York University Tandon School of Engineering Broonlyn New York USA

**Keywords:** epidemic control, epidemic modeling, model predictive control, nonlinear modeling, nonlinear optimization

## Abstract

The COVID‐19 pandemic has led to the unprecedented challenge of devising massive vaccination rollouts, toward slowing down and eventually extinguishing the diffusion of the virus. The two‐dose vaccination procedure, speed requirements, and the scarcity of doses, suitable spaces, and personnel, make the optimal design of such rollouts a complex problem. Mathematical modeling, which has already proved to be determinant in the early phases of the pandemic, can again be a powerful tool to assist public health authorities in optimally planning the vaccination rollout. Here, we propose a novel epidemic model tailored to COVID‐19, which includes the effect of nonpharmaceutical interventions and a concurrent two‐dose vaccination campaign. Then, we leverage nonlinear model predictive control to devise optimal scheduling of first and second doses, accounting both for the healthcare needs and for the socio‐economic costs associated with the epidemics. We calibrate our model to the 2021 COVID‐19 vaccination campaign in Italy. Specifically, once identified the epidemic parameters from officially reported data, we numerically assess the effectiveness of the obtained optimal vaccination rollouts for the two most used vaccines. Determining the optimal vaccination strategy is nontrivial, as it depends on the efficacy and duration of the first‐dose partial immunization, whereby the prioritization of first doses and the delay of second doses may be effective for vaccines with sufficiently strong first‐dose immunization. Our model and optimization approach provide a flexible tool that can be adopted to help devise the current COVID‐19 vaccination campaign, and increase preparedness for future epidemics.

## 1 INTRODUCTION

Since its inception in December 2019, COVID‐19 has rapidly become a global pandemic, infecting more than 190 million people, with more than 4 million fatalities as of July 2021.^1^ As a response to such a global health crisis, pharmaceutical researchers made an extraordinary efforts toward the development of effective vaccines against the novel disease,^2^, ^3^, ^4^, ^5^ and most countries are currently undergoing a vaccination campaign.^6^ The first vaccines developed and used in the vaccination campaigns require the administration of two doses to be injected within an interval of few weeks (typically, 3–12, depending on the vaccine and on the local vaccination policies).^7^, ^8^, ^9^, ^10^ However, different from standard vaccines and drugs for which the approval by public pharmaceutical agencies is typically a long procedure, the current health crisis has called for the implementation of extraordinary fast approval procedures. As a consequence, an agreement among public health authorities and researchers on a common protocol on vaccination strategies and delays between the two doses has not been found yet.^9^, ^10^

Mathematical models of epidemic spreading have emerged as a valuable paradigm to predict the spread of epidemic diseases and assess the effectiveness of different intervention policies.^11^, ^12^, ^13^, ^14^, ^15^ Notably, in response to the COVID‐19 pandemic, several mathematical models have been developed,^16^, ^17^, ^18^, ^19^, ^20^, ^21^, ^22^, ^23^, ^24^, ^25^, ^26^, ^27^ with the aim of supporting decision makers in the implementation of nonpharmaceutical interventions (NPIs) during the first phases of the outbreak in 2020,^20^, ^21^, ^22^, ^23^ and on their gradual uplifting during the 2021 vaccination campaigns.^24^, ^25^, ^26^, ^27^

Besides predicting the spread of the disease and assessing the effectiveness of NPIs, mathematical models can also provide valuable insight to assist vaccination campaigns. Specifically, the systems and controls approach to epidemic modeling has provided powerful tools to study how to optimally distribute drugs and vaccines in a population by formalizing and solving resource allocation problems.^28^, ^29^, ^30^ More details can be found in recent review papers.^12^, ^15^ However, all these approaches rely on the simplifying assumption that the vaccination procedure consists in a single dose, after which individuals become immune to the disease, while more complex and realistic vaccination procedures (including the multidose procedures that characterize most of the COVID‐19 vaccines)^7^, ^8^ have often been overlooked. Motivated by the current challenges, some efforts have been recently proposed to assess the effectiveness of two‐dose vaccination rollouts.^31^, ^32^ However, to the best of our knowledge, mathematical tools to optimally design a two‐dose vaccination rollout are still missing.

In this article, we fill in this gap by proposing a methodological approach to optimally calibrate a two‐dose vaccination strategy during an epidemic outbreak, based on nonlinear model predictive control (MPC).^33^, ^34^ First, we propose a mathematical epidemic model tailored to the COVID‐19 progression. Specifically, we extend a discrete‐time, deterministic susceptible–infected–recovered (SIR) population model,^13^ by adding further compartments to represent different stages of the disease progression and of the two‐dose vaccination procedure, encompassing a delay between the first and the second dose and a partial immunity of limited duration that can be gained after the first dose. Then, we utilize nonlinear MPC to optimally design the vaccination rollout, that is, to plan the scheduling of first and second doses for the entire duration of the vaccination campaign. The nonlinear optimization problem underlying the MPC has for objective the concurrent minimization of both the healthcare impact of the epidemic and of the socio‐economic impact due to the implementation of NPIs. The epidemic model and its related optimization strategy are easily adaptable to any airborne disease with similar vaccination characteristics.

We calibrate the model on the 2021 vaccination campaign against COVID‐19 in Italy. Specifically, we calibrate the epidemic model using the officially reported epidemiological data during the “second wave” of the COVID‐19 Italian outbreak (from September 2020 to March 2021).^35^ Then, we use nonlinear MPC to devise the optimal vaccination strategy for the two vaccines that were most used in Italy during the first phase of the vaccination rollout: Comirnaty (BNT162b2) by Pfizer–BioNTech and Vaxzevria (ChAdOx1‐S) by AstraZeneca, for which we have estimated the corresponding model parameters from clinical data.^2^, ^3^, ^4^, ^5^

Our findings confirm that optimizing the vaccination rollout is a nontrivial problem.^9^, ^10^ In fact, the optimal solution depends on the characteristics of the vaccine—namely, on the level and duration of the partial immunity entailed by the first‐dose—and on the spread of the epidemics. Specifically, we find that the first dose can be prioritized in the early stages of the rollout for vaccines with a sufficiently efficacious first dose and a long duration of the partial immunity provided (e.g., AstraZeneca).^5^ This prioritization entails a (partial) herd immunity that is effective in avoiding resurgent waves. On the contrary, for vaccines with a lower first‐dose efficacy and a shorter minimum delay between the doses, an alternate vaccination strategy that minimizes the delay between the first and the second dose may be preferable, toward keeping a uniform level of immunity over the vaccinated population. Our analysis also highlights the flexibility of our approach, which enables to test several what/if scenarios, demonstrating its potential use not only to help assist public health authorities in their current decisions on planning the COVID‐19 vaccination campaigns, but also to create preparedness for future pandemics.

The rest of the article is organized as follows. In Section 2, we propose the mathematical epidemic model and formalize the optimization problem. In Section 3, we calibrate the model to the COVID‐19 outbreak in Italy. Section 4 contains our main findings. Section 5 concludes the article and outlines avenues for future research.

## 2 MODEL

In this section, we present the mathematical model for the disease progression and vaccination dynamics, and we formalize the optimal vaccination rollout problem.

### 2.1 Epidemic model

We propose an extension of a discrete‐time, deterministic SIR population model,^13^ which accounts for (i) different stages and outcomes of the disease, including hospitalization, recovery, and death; (ii) a time‐varying infection rate that captures the effect of the implementation of NPIs; and (iii) a two‐dose vaccination procedure, with a fixed minimum interval between the two doses, and partial immunity gained after the first dose.

**FIGURE 1:**
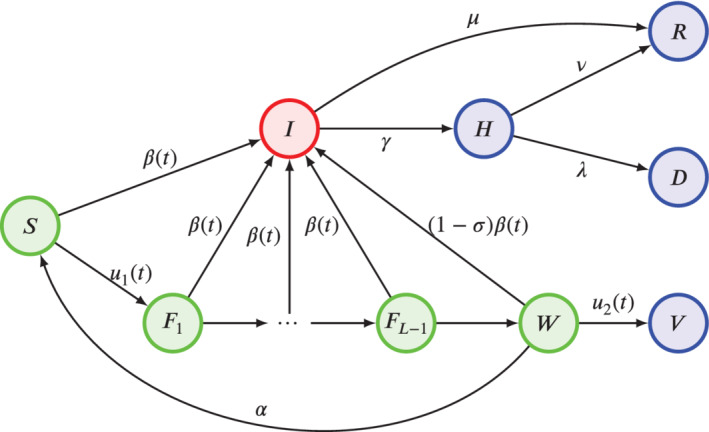
Schematic of the epidemic process. Colors are used to denote different infectiousness statuses. Green nodes are susceptible to the disease (with those in *W* with a reduced risk due to partial immunity); the red node denotes individuals that are infectious; blue nodes are individuals that are nor infectious nor susceptible (because recovered, dead, isolated, or fully vaccinated)

We denote the discrete time variable ad $t\in {\mathbb{Z}}_{\geq 0}$ and we set the discrete time unit equal to one week. Similar to a standard SIR model, a population of *N* individuals is partitioned into a set of compartments that represent the possible health and vaccination states of the individuals. With respect to a standard SIR, our model incorporates some further compartments described in the following and shown in Figure 1.

For the disease progression, we consider five different compartments. Specifically, we denote with S(t) the number of *susceptible* individuals in the population at time *t*, with I(t) the number of the *infectious* ones (with no distinctions between symptomatic and asymptomatic), with H(t) the number of *hospitalized* ones, with R(t) the number of the *recovered* ones, and with D(t) the number of *dead* ones. Different from many other models tailored to COVID‐19,^16^, ^20^, ^21^ our formulation does not require the introduction of an intermediate compartment between contagion and infectiousness (often termed “exposed”). In fact, in our model, such a delay is naturally incorporated within the discrete‐time modeling approach with weekly time‐steps, which implies that, upon contagion, individuals become infectious only at the following time‐step. Such a one‐week delay is compatible with the average progression dynamics of COVID‐19.^36^ However, the proposed model is of general validity and the approach can be easily extended to accommodate for longer durations of the “exposed” state by adding further compartments.

The epidemic process is modeled through the following dynamics, which take place concurrently.

Contagion The contagion dynamics causes a fraction of the susceptible individuals to become infected and to transition from compartment *S* to *I* at time t+1. Such a fraction is proportional to the fraction of infectious individuals in the population, that is, I(t)/N, and to a time‐varying *infection rate* β(t)≥0, which captures the transmissibility of the disease and the effect of NPIs in reducing human‐to‐human contacts through which the disease spreads.
Recovery Among the infectious individuals I(t), a fraction μ∈[0,1] of them recovers and transitions to compartment *R* at time t+1.
Hospitalization Among the infectious individuals I(t), a fraction γ∈[0,1] is hospitalized, transitioning to the compartment *H* at the following time‐step.
Hospital discharge Among the hospitalized individuals, a fraction ν∈[0,1] recovers and is discharged from the hospital, transitioning to R(t+1).
Death Among the hospitalized individuals, a fraction λ∈[0,1] dies, transitioning to D(t+1).

We assume that recovered individuals cannot be infected again for the duration of our simulation, which is consistent with the clinical literature that founds evidence that immunity lasts at least 6–8 months.^37^, ^38^

The vaccination dynamics is modeled by adding further compartments to the model. We consider a two‐dose vaccination procedure, where the two doses must be inoculated a minimum of *L* weeks apart (for instance, for the COVID‐19 vaccines, 3 weeks is the minimum interval between the two doses for Pfizer–BioNTech vaccine,^7^ while 4 weeks is the minimum for AstraZeneca vaccine).^8^ Hence, we consider L−1 additional compartments to represent individuals that were *vaccinated with the first dose* in the previous week ($F_{1}(t)$), two weeks before ($F_{2}(t)$), up to L−1 weeks before ($F_{L-1}(t)$). Then, we add one compartment to account for individuals that were vaccinated with the first dose at least *L* weeks before, and thus are *ready to receive the second dose*, denoted by W(t), and one for individuals that have received the second dose and are *fully vaccinated*, denoted as V(t).

At time *t*, both first and second doses may be administered to the population. Specifically, $u_{1}(t)$ susceptible individuals receive their first dose, thus transitioning from state *S* to state $F_{1}$ at the following time‐step (week); and $u_{2}(t)$ individuals who are ready for full vaccination receive their second dose, thus transitioning from *W* to *V* at the following time‐step (week). We assume that individuals who have received both doses are fully immunized against COVID‐19 and cannot be infected for the time of our simulations, while those who have received only one dose can still be infected at a reduced rate. Specifically, we assume that individuals in compartment $F_{t}(t)$, with t=1,…,L−1, have the same infection risk of susceptible individuals. Hence, a fraction β(t)I(t)/N of them becomes infected at each time‐step and transitions to I(t+1), while the rest transition through a cascade mechanism, from $F_{1}$ to $F_{2}$, from $F_{2}$ to $F_{3}$, and so forth, up to those in $F_{L-1}$ who transition to *W*. We assume that those who are waiting for the second dose W(t) have a partial immunity, as supported by clinical data.^2^, ^3^, ^4^ Hence, the infection rate for these individuals is reduced by a parameter σ∈[0,1], which models the *partial immunization* caused by the first dose, while only a fraction (1−σ)β(t)I(t)/N of them transitions to the infectious state. Finally, we assume that the efficacy of the partial immunization guaranteed by the first dose has a limited duration, after which its effects are lost and the individual becomes again susceptible to the disease (with no partial immunity). We model this phenomenon by assuming that, at each time‐step, a fraction α∈[0,1] of individuals in W(t) transitions back to the susceptible state *S*, where 1/α is the average duration of the partial immunization.

The time‐varying infection rate β(t) is designed to mimic the evolution of the implementation of NPIs during the course of the epidemic.^39^, ^40^ Specifically, we set $\beta (t)=\beta_{0}\phi (t)$, where $\beta_{0}$ is the infection rate in the absence of severe NPIs and ϕ(t) is a time‐varying function that captures the reduction of social activity due to the implementation of NPIs (where ϕ(t)=1 is the maximum level of social activity). Since NPIs are typically enforced and strengthened when the number of infections and hospitalizations increases,^41^ we let ϕ(t) evolve according to a feedback mechanism based on the size of the hospitalized compartment, similar to some recently proposed feedback‐controlled SIR models.^42^, ^43^ First, we define a lower‐bound value for social activity, corresponding to the maximum level of NPIs as $\phi_{\text{NPI}}\in [0,1)$. Then, we let the social activity vary continuously between this value and 1 (which corresponds to the absence of severe NPIs), according to

(1) $$\phi (t+1)=\phi_{\text{NPI}}+(1-\phi_{\text{NPI}})\left(1+\frac{1}{2}\tanh\left(\kappa \frac{H(t)-\bar{H}}{\bar{H}}\right)\right)\;,$$

where κ>0 is the *reactivity* of the population to changes in NPIs, which determines the velocity of adoption of behaviors that reduce the contagions when NPIs are implemented and the velocity of returning to normalcy upon their uplifting, and $\bar{H}$ is a *critical number of hospitalization*. Finally, (1) yields the following expression for the time‐varying infection rate:

(2) $$\beta (t+1)=\beta_{0}\left(\phi_{\text{NPI}}+(1-\phi_{\text{NPI}})\left(1+\frac{1}{2}\tanh\left(\kappa \frac{H(t)-\bar{H}}{\bar{H}}\right)\right)\right)\;.$$

As we shall discuss in the following section, we will identify the four parameters $\beta_{0},\phi_{\text{NPI}},\kappa$, and $\bar{H}$ from available epidemic data.^35^

Finally, we define a variable B(t) that quantifies the number of vaccine doses available in stock at time *t*. Hence, $B(t+1)=B(t)+Y(t)-u_{1}(t)-u_{2}(t)$, where Y(t) are the new doses delivered at time *t*. Note that, in general Y(t) can be a deterministic sequence, or a realization of stochastic variables that may capture the possible uncertainties in the deliveries.

When the population size *N* is large, we can approximate the state variables as continuous, as typically assumed in mathematical epidemic models,^14^, ^15^ and define the following (8+L)‐dimensional system of recurrence equations, which governs the epidemic dynamics:

(3) $$\begin{array}{ll} & S(t+1)=S(t)-\beta (t)S(t)\frac{I(t)}{N}+\alpha W(t)-u_{1}(t)\\ & I(t+1)=(1-\mu -\gamma )I(t)+\beta (t)\left(S(t)+\overset{L-1}{\underset{\ell =1}{\sum }}F_{\ell }(t)+(1-\sigma )W(t)\right)\frac{I(t)}{N}\\ & H(t+1)=(1-\nu -\lambda )H(t)+\gamma I(t)\\ & R(t+1)=R(t)+\mu I(t)+\nu H(t)\\ & D(t+1)=D(t)+\lambda H(t)\\ & F_{1}(t+1)=u_{1}(t)\\ & F_{\ell }(t+1)=F_{\ell -1}(t)\left(1-\beta (t)\frac{I(t)}{N}\right),\;\;\ell =2,\ldots ,L-1\\ & W(t+1)=(1-\alpha )W(t)+F_{L-1}(t)\left(1-\beta (t)\frac{I(t)}{N}\right)-(1-\sigma )\beta W(t)\frac{I(t)}{N}-u_{2}(t)\\ & V(t+1)=V(t)+u_{2}(t)\\ & \beta (t+1)=\beta_{0}\left(\phi_{\text{NPI}}+(1-\phi_{\text{NPI}})\left(1+\frac{1}{2}\tanh(\kappa \frac{H(t)-\bar{H}}{\bar{H}})\right)\right)\\ & B(t+1)=B(t)+Y(t)-u_{1}(t)-u_{2}(t)\;, \end{array}$$

where the sequence of deliveries Y(0),Y(1),… is assumed to be known (deterministically, or as a random variable) and the variables $u_{1}(t)$ and $u_{2}(t)$ are the two control inputs.

Remark 1 We briefly comment that this modeling framework can be straightforwardly adapted to capture further real‐world features of vaccination. For instance, imperfect efficacy of full vaccination can be incorporated by simply adding a possible transition between compartment *V* and compartment *I*, with an opportunely reduced infection rate. Moreover, our model relies on the simplifying assumption that partial immunity is gained only *L* weeks after the first dose, that is, when individuals reach state *W* . Such an assumption can be relaxed, by introducing a parameter $\sigma_{\ell }$ that captures the partial immunity gained ℓ weeks after the first dose, and opportunely rescaling the infection rate β(t).

Remark 2 Note that, in the epidemic model in (3), the effective reproduction number $R_{t}$ can be computed following its definition as the average number of secondary infections generated by an infected individual as

(4) $$R_{t}:=\frac{\beta (t)}{\mu +\gamma }\frac{S(t)}{N}\;.$$

### 2.2 Optimization problem

In this section, we provide the details of the optimization approach that we use to devise the optimal vaccination rollout, based on nonlinear MPC.^33^, ^34^ In particular, our goal is to understand how to optimally administer the two doses $u_{1}(t)$ and $u_{2}(t)$ along the entire vaccination campaign, in order to keep the number of hospitalizations under control and reduce the need of implementing NPIs. Hence, fixing the time‐horizon of the optimization process $T\in {\mathbb{Z}}_{>0}$, which coincides with the entire duration of the vaccination rollout, the control variable is defined as a 2T‐dimensional vector $u=(u_{1}(0),\ldots ,u_{1}(T-1),u_{2}(0),\ldots ,u_{2}(T-1))$. To keep the notation compact, we will denote by x the vector that gathers all the variables of the dynamical system in (3) from t=1 to t=T.

#### 2.2.1 Cost function

The goal of designing an optimal vaccination rollout is to quickly achieve herd immunity while, first, keeping low the pressure on the healthcare system (captured by the number of hospitalized individuals H(t)) and second, allowing the relaxation of NPIs, thereby allowing a fast return to normalcy. In order to model these two competing targets, we design a quadratic cost function J(u,x) that accounts for two distinct factors, as

(5) $$J(u,x)=\overset{T}{\underset{i=1}{\sum }}{\left(\frac{H(t)}{H_{\max}}\right)}^{2}+\overset{T}{\underset{t=1}{\sum }}{\left(\frac{1-\phi (t)}{1-\phi_{\text{NPI}}}\right)}^{2}.$$

The first term in (5) captures the *healthcare cost* and is proportional to the sum of the squares of the number of hospitalized H(t) individuals, along the entire duration of the vaccination campaigns.

The second term, instead, considers the *socio‐economic cost* associated with the implementation of NPIs and the current reduction of social and economical interactions among the individuals in the population (e.g., due to the enforcement of social distancing, curfews, or closures of nonessential economic activities). This term sums the squares of the discrepancy between the actual value of the individual social activity ϕ(t) and its desirable value in the absence of severe NPIs (i.e., 1).

The two terms are then properly weighted to evenly balance the two contributions. In particular, in the healthcare cost, the number of hospitalized H(t) is divided by $H_{\max}=37,383$ representing the maximum number of hospitalized individuals obtained through simulating the scenario without vaccination. As a result each summand of the healthcare cost is bounded between [0,1]. The socio‐economic cost, instead, is divided by $1-\phi_{\text{NPI}}$ that represents the maximum discrepancy between the social activity ϕ(t) and its value in the absence of severe NPIs. Hence, also in the second term, each summand is bounded between [0,1].

#### 2.2.2 Constraints

Here, we provide the details of the additional constraints that the optimal solution has to meet, besides verifying the dynamical system in (3). In particular, the control variable u(t) needs to satisfy constraints on the total number of weekly doses injected, which cannot be more than the healthcare capacity, nor more than the available doses. Furthermore, the number of first and second doses inoculated each week cannot be greater than the number of individuals that are admissible to receive them (i.e., S(t) and W(t), respectively). All these constraints are gathered in the following list:

- We set an upper‐bound *U* on the total number of doses that is possible to inoculate in one week. This accounts for the maximum capacity of the healthcare system to accommodate vaccinations. Hence, we set the constraint
(6) $$u_{1}(t)+u_{2}(t)\leq U.$$
- The constraint B(t)≥0 is set to guarantee that the number of doses done in week *t* does not exceed the number of vaccines available in stock at time *t*.
- The constrain S(t)≥0 is enforced to guarantee that the number of first doses $u_{1}(t)$ is not greater than the number of susceptible individuals, available for the vaccination campaign.
- Finally, the constrain W(t)≥0 is enforced to ensure that the number of second doses performed each week $u_{2}(t)$ is not larger than the individuals admissible for a second dose.

We would like to comment that some additional constraints may be considered. In particular, one may want to enforce that the number of hospitalizations H(t) do never trespass a certain threshold, which may depend on the healthcare capacity. In our implementation, we have decided to omit this constraint, since the feedback mechanism that regulates the time‐varying infection rate β(t) in (2) implicitly induces an upper‐bound on the total number of hospitalizations, which can be opportunely calibrated by setting the parameters in (2).

#### 2.2.3 Formulation of the optimization problem in the MPC framework

Based on the considerations above, we formalize the optimal vaccination problem through the following minimization problem:

(7) $$\begin{array}{rll} \text{minimize}\; & \left(5\right) & \\ \text{subject to}\; & \left(3\right) & \\ & u_{1}(t)\geq 0, & \;\forall t\in \{0,\ldots ,T-1\},\\ & u_{2}(t)\geq 0, & \;\forall t\in \{0,\ldots ,T-1\},\\ & u_{1}(t)+u_{2}(t)\leq U, & \;\forall t\in \{0,\ldots ,T-1\},\\ & B(t)\geq 0, & \;\forall t\in \{1,\ldots ,T\},\\ & S(t)\geq 0, & \;\forall t\in \{1,\ldots ,T\},\\ & W(t)\geq 0, & \;\forall t\in \{1,\ldots ,T\}. \end{array}$$

In general, the solution of the minimization problem in (7) is nontrivial, due to the complex and nonlinear structure of the dynamical system in (3). Indeed, the problem is not trivial even if we neglect the presence of the contagion process and we make the oversimplification that the optimal vaccination strategy is the one that maximizes the number of protected individuals for the entire duration of the process. In fact, apart from the limit case σ=1 and α=0, in which the optimal solution would be to perform just first doses ($u_{1}(t)=U$, for all t=0,…,T−1), it is in general not possible to find a unique solution that maximizes the number of protected individuals at all the time‐steps, since different solutions may yield maximal protections for different time‐instances. The complexity of the problem becomes more evident when the nonlinear epidemic process is also taken into account, whereby the nonmonotonicity of the dynamical system in (3), its dependence on the initial condition and on the implementation of NPIs hinders the direct analytical treatment of the minimization problem and the derivation of analytical insights.

In this work, the nonlinear optimization problem in (7) is solved within the MPC framework.^34^ Specifically, the solution of the nonlinear MPC is based on iterative, finite‐horizon optimization of the cost function J(u,x). At time *t*, the current state is sampled and a cost‐minimizing control strategy is computed via a numerical minimization algorithm for a time horizon of 15 weeks. Only the first step of the control strategy is executed, and then the model evolves to a new state at time t+1. The calculations are repeated starting from the new current state, yielding a new sample of the control input and a new instance of the predicted state.

## 3 CALIBRATION TO THE 2021 COVID‐19 VACCINATION CAMPAIGN IN ITALY

We calibrate the model to reproduce the Italian outbreak of COVID‐19, identifying the epidemiological parameters from publicly available epidemiological data.^35^ Then, we calibrate the parameters related to the vaccination campaign by using clinical data on the two mostly used vaccines during the first phases of the vaccination campaign, that is, the Pfizer–BioNTech and the AstraZeneca COVID‐19 vaccines.

### 3.1 Calibration of the epidemic model

We identify the epidemiological parameters from data on the number of hospitalizations and deaths, reported by the official Italian Civil Protection Department (*Dipartimento della Protezione Civile*) in a publicly available database.^35^ Namely, we identify the following four transition rates: μ, from *I* to *R*; γ, from *I* to *H*; ν, from *H* to *R*; and λ, from *H* to *D*. Moreover, we identify the four parameters governing the dynamics of the infection rate in (2), that is, the rate in the absence of severe NPIs $\beta_{0}$, the activity reduction in the presence of severe NPIs bounds $\phi_{\text{NPI}}$, the critical number of hospitalizations $\bar{H}$, and the population reactivity to changes in the NPIs κ.

We execute the calibration on epidemic data collected between September 1, 2020 and March 23, 2021 (for a total of 29 weeks). This time‐window spans from the inception of the second‐wave to the third wave in Italy, which is still ongoing as of May 11, 2021. In the selected time‐window, vaccinations had a negligible effect. In fact, even though the vaccination rollout in Italy officially started on December 27, 2020, less than 4.5% of the population was fully vaccinated as of March 23, 2021.^6^ Hence, to identify the epidemiological parameters of the model, we consider a pertinent submodel (i.e., SIRHD), obtained from the first five equations of (3) and by setting $u_{1}(t)=u_{2}(t)=0$, for all $t\in {\mathbb{Z}}_{\geq 0}$, and $F_{1}(0)=\ldots =F_{L-1}(0)=W(0)=V(0)=0$.

The model is initialized with the official data on the number of hospitalized individuals, cumulative recovers, and cumulative deaths as of September 1, 2020,^35^ which allows us to set H(0)=1,686 and D(0)=35,520, while we assume that the rest of the population is either susceptible or infected. Since the official number of reported infections is subject to possible underreporting due to the presence of asymptomatic carriers and delays and a reliable estimations of its initial value I(0) is not available, such initial condition is thus set as a parameter $I_{0}$ to be identified. Finally, we set S(0)=N−I(0)−H(0)−D(0), where N=59,394,207 is the total Italian population, from Census data.^44^

The parameter identification is performed by solving a suitable minimization problem. Specifically, we define a cost function *C* as

(8) $$C=\overset{T_{\text{id}}}{\underset{t=1}{\sum }}{\left(H(t)-Ĥ(t)\right)}^{2}+\overset{T_{\text{id}}}{\underset{t=1}{\sum }}{\left(D(t)-\hat{D}(t)\right)}^{2}\;,$$

where $T_{\text{id}}$ is the duration of the time‐window of the parameter identification, that is, $T_{\text{id}}=29$ weeks. The cost function *C* is formed by two terms. The former accounts for the sum of the squared errors between the number of hospitalizations (H(t)) predicted by the model and those officially reported (denoted by Ĥ(t)), aggregated at a weekly level.^35^ The latter is similar and accounts for the errors between the deaths predicted by the model (D(t)) and those officially reported ($\hat{D}(t)$). Hence, the nine model parameters are estimated as

(9) $$(\mu^{*},\gamma^{*},\nu^{*},\lambda^{*},\beta_{0}^{*},\phi_{\text{NPI}}^{*},{\bar{H}}^{*},\kappa^{*},I_{0}^{*})=\underset{\mu ,\gamma ,\nu ,\lambda ,\bar{H},\beta_{0},\phi_{\text{NPI}},\kappa ,I_{0}}{\text{argmin}}C.$$

**FIGURE 2:**
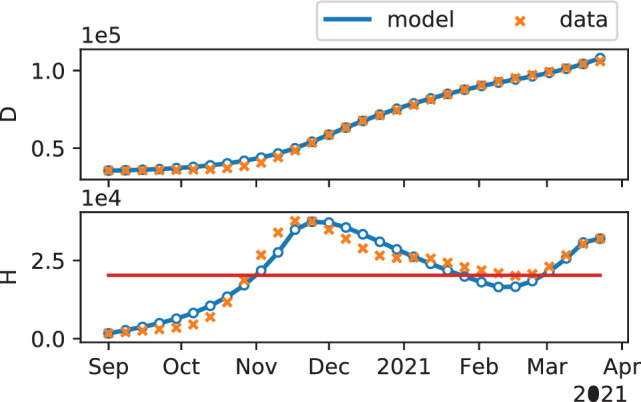
Results of the model calibration. The first two plots show the temporal evolution of the number of deaths and hospitalized individuals, respectively, as predicted by the model (blue curve, where a series of resurgent waves can be noticed), compared with the officially reported data (orange crosses) that are used to calibrate the model.^35^ The red horizontal line in the second plot denotes the estimated critical number of hospitalizations $\bar{H}$. The model parameters identified are summarized in Table 1

The minimization problem in (9) is solved numerically by means of a dual‐annealing procedure,^45^ yielding the parameters reported in Table 1. Figure 2 shows the results of the numerical calibration procedure. Note that, in the absence of severe NPIs, our calibration procedure estimates that the reproduction number in a population of susceptible individuals would tend to 1.65 (see Remark 2), whereas severe NPIs would always reduce it to below 1.

**TABLE 1:** Model parameters identified by fitting Italian official data of reported hospitalizations and deaths^35^

Symbol	Meaning	Value
μ	transition rate I→R	0.488
γ	transition rate I→H	0.0279
ν	transition rate H→R	0.356
λ	transition rate H→D	0.122
$\phi_{\text{NPI}}$	social activity reduction with severe NPIs	0.572
$\beta_{0}$	infection rate without severe NPIs	0.799
$\bar{H}$	critical number of hospitalizations	20,320
κ	reactivity of the population	20.32

Abbreviation: NPI, nonpharmaceutical intervention.

### 3.2 Vaccine parameters

We utilize clinical data to set the three parameters that characterize the specific features of the vaccines under consideration. Specifically, we consider the two vaccines that were mostly used in Italy during the first phase of the vaccination campaign: Comirnaty (BNT162b2) by Pfizer–BioNTech and Vaxzevria (ChAdOx1‐S) by AstraZeneca. For the sake of simplicity, we will refer to each vaccine using the name of the pharmaceutical company that has developed it, that is, Pfizer–BioNTech and AstraZeneca, respectively. The three parameters, namely, the minimum number of weeks between the first and the second dose *L*, the efficacy of the first dose σ, and the velocity of loss of partial immunity α, are set as detailed in the following and summarized in Table 2.

Pfizer–BioNTech. The minimum interval between the two doses for Pfizer–BioNTech vaccine was identified as L=3 weeks via clinical studies by Pfizer.^7^ The efficacy of a single dose, in “Phase 3” clinical trials, during the interval between first and second doses was estimated at 52% (95% confidence interval [30%, 68%]).^2^ Another recent study analyzing the vaccination campaign in Israel, confirm the efficacy against PCR‐confirmed similar results (14–20 days after the first dose 46% (95% confidence interval [40%, 51%]) and 21–27 after the first dose 60% (95% confidence interval [53%, 66%]).^3^ We set for our analysis σ=0.52.
AstraZeneca. We set L=4 weeks, which is the minimum for interval between the two doses of AstraZeneca vaccine determined in the clinical trials^8^). Clinical data of the efficacy of a single dose of the AstraZeneca vaccine are reported from different places in the world (UK, Brazil, and South Africa), confirming a vaccine efficacy of 76% (95% confidence interval [59%, 86%]) after a first dose, with protection maintained to the second dose.^5^ Accordingly, we set σ=0.76.

**TABLE 2:** Parameters of the two vaccines in the case study

Symbol	Meaning	Pfizer–BioNTech	AstraZeneca
*L*	minimum weeks from the first to the second dose	3	4
σ	efficacy of first dose	0.52	0.76
α	velocity of loss of partial immunity	0.0833	0.0833

At the time being, α, accounting for the vanishing of the first dose immunity, is still unknown or vaguely guessed from the literature for both vaccines.^3^, ^5^ As a consequence, we initially make an educated guess by setting α=1/12, as 12 weeks is the maximum duration of the period between two doses tested in clinical trials. In Section 4.3, we will then explore the effect of different values of α on the optimal vaccination rollout.

## 4 RESULTS

In this section, we study the problem of the two‐dose vaccination rollout with the two vaccines, namely, Pfizer–BioNTech and AstraZeneca (characterized by the parameters reported in Table 2). In our analysis, we focus on a year‐long vaccination campaign (T=52 weeks), starting from week 52 of 2020 (December 21–28), that is, the first week of the Italian vaccination campaign.^46^ To this aim, we initialize the epidemic model in (3) with I(0)=597,719, H(0)=33,450, D(0)=67,631, S(0)=55,211,502, and $F_{1}(0)=\ldots =F_{L-1}(0)=W(0)=V(0)=0$ (with L=3 for Pfizer–BioNTech and L=4 for AstraZeneca), as estimated from our calibrated model. Note that we have opted to initialize the system with the conditions generated by our model (calibrated on real‐world data, see Table 1) instead of using directly the reported data, to avoid the possible underestimation of the number of infected individuals due to underreporting. In our model, we consider a regular (deterministic) weekly supply of Y(t)=1,000,000 doses for each $t\in {\mathbb{Z}}_{\geq 0}$, an amount that is consistent with the average weekly doses delivered in the period between February and May 2021 in Italy.^46^ Finally, when needed, the weekly upper bound for the total number of injection $u_{1}(t)+u_{2}(t)\leq U=5,000,000$ doses, consistent with the limitations of the healthcare system, when at full capacity.^46^

### 4.1 A comparison between two trivial strategies

We start our analysis by presenting a motivating example that illustrates the complexity of the optimization problem. In particular, we consider two simplified vaccination strategies consisting in minimizing the delay between the two doses and prioritizing the first doses, respectively. These two strategies are explicitly defined in the following.

Alternating strategy This solution aims at maximizing the total number of fully vaccinated individuals, by alternating first and second doses. Specifically, all the individuals that receive the first dose during week *t*, and not get infected while in, will receive the second dose exactly during week t+L. Accordingly, at each time step the control u(t) is given by:
(10) $$\left\{\begin{array}{c} u_{1}(t)=Y(t)-u_{2}(t)\\ u_{2}(t)=W(t)\;, \end{array}\right.\;$$
  where we observe that the constraint $u_{1}(t)+u_{2}(t)\leq U$ is always verified, since Y(t)<U.
First doses only In this case all the available doses are used for the first doses, that is,
(11) $$\left\{\begin{array}{c} u_{1}(t)\;=Y(t)\\ u_{2}(t)=0. \end{array}\right.\;$$

We analyze these two different strategies in terms of the cost *J* defined in (5), with the purpose of understanding the circumstances in which one strategy is more efficient than the other. Specifically, we compare the two strategies for the two vaccines, and for different values of α, for which there is still uncertainty among the scientific community.^3^, ^5^

**FIGURE 3:**
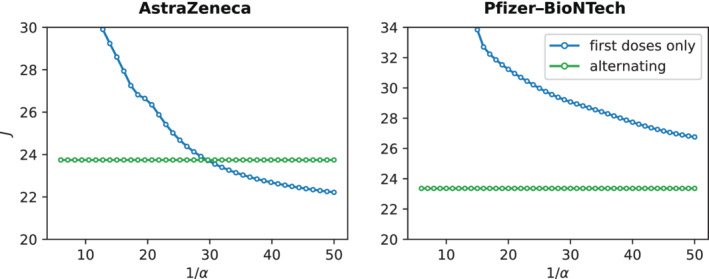
Comparison between the two trivial strategies. The figure shows the cost *J* defined in (5) for the *alternating strategy* (green curve) and the *first doses only strategy* (blue curve) defined in Section 4.1, for different values of the velocity of loss of partial immunity α. The left plot shows the results for the AstraZeneca vaccine, the right one for Pfizer–BioNTech

The results for the two types of vaccine, reported in Figure 3, show a nontrivial behavior of the cost function. While for the Pfizer–BioNTech vaccine, the alternating strategy seems to always outperform the first doses prioritization one, AstraZeneca shows a different behavior, whereby the latter becomes preferable when α<1/29.

The analysis of these two trivial strategies confirms the complexity of the problems at hand, where model parameters may play an important role. In the rest of this section, we will utilize our MPC‐based strategy to derive the structure of the optimal vaccination policy and shed light on its dependencies on the characteristics of the vaccine in use.

### 4.2 Optimal vaccination rollout for Pfizer–BioNTech and AstraZeneca

**FIGURE 4:**
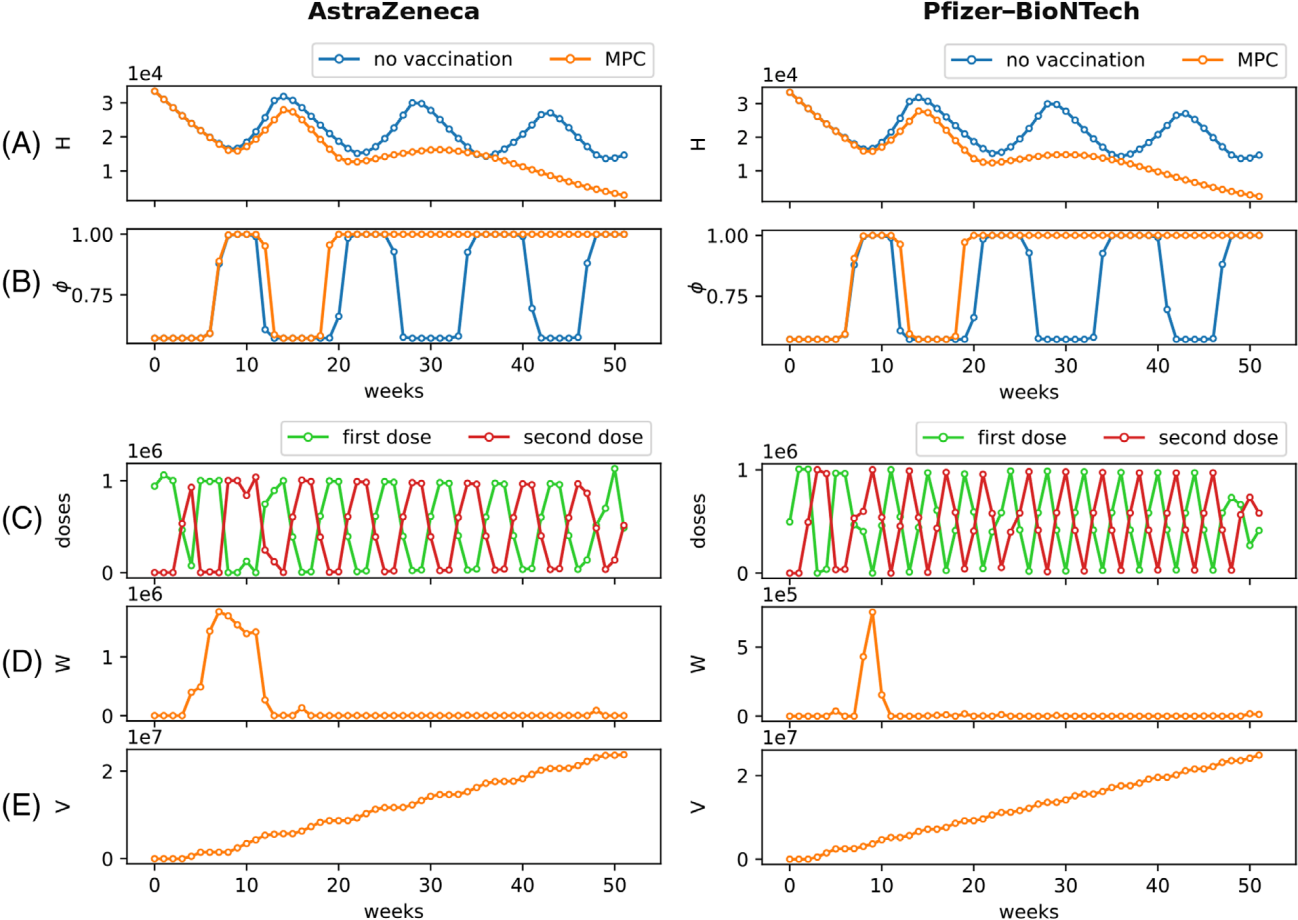
Optimal vaccination rollout. On the left, we show the results of the MPC for AstraZeneca, on the right those for Pfizer–BioNTech (see Table 1). In (A), we compare the temporal evolution of the number of hospitalized individuals H(t) without vaccinations (blue curve) and implementing the optimal strategy proposed by the MPC (orange curve). In (B), we compare the temporal evolution of social activity ϕ(t) as an indication of the timeline of NPIs in the absence (blue curve) and presence (orange curve) of vaccination. The panels in (C) illustrate the number of first doses (green) and second doses (red) that should be performed each week, under the optimal rollout computed using the MPC. The temporal evolutions of the number of individuals who are ready to receive the second dose, but for whom the second dose is delayed W(t), and of the number of fully vaccinated individuals V(t) are reported in panels (D) and (E), respectively. MPC, model predictive control; NPI, nonpharmaceutical intervention

We now examine the optimal vaccination rollout strategies for the two vaccines, obtained by solving the optimization problem in (7) by means of nonlinear MPC.^34^ Our findings are summarized in Figure 4. First, we evaluate the effectiveness of the vaccination rollout that employs the MPC solution in slowing down the epidemic and reducing the socio‐economic costs associated with lockdowns. In Figure 4(A,B), we start by comparing the temporal evolution of the system in the presence and in the absence of the vaccination campaign. From these figures, we immediately observe that vaccination is key to decreasing the number of hospitalizations and avoid the need of multiple lockdowns. In fact, from week 20 (when about 19% of the population is fully vaccinated), the evolution of the system with vaccination (orange curve) starts diverging sensibly from the system in the absence of it (blue curve) and it does not require further implementation of severe NPIs, with both vaccines.

The optimal vaccination rollouts are reported in Figure 4(C). For both the vaccines, the optimal strategies tend to distribute evenly first and second doses, creating an alternation between the two doses. In addition all the doses are immediately used (see Figure 4(D,E)). However, how to optimally design such an alternation policy is indeed nontrivial. In fact, while for the AstraZeneca vaccine it seems that the optimal strategy alternates weeks in which (almost) only first doses are performed and weeks with (almost) only second doses, for the Pfizer–BioNTech vaccine, after a very short transient, the optimal vaccination strategy seems to require an (approximately) similar number of first and second doses each week, with small periodic oscillations.

Further analysis of the solutions shows that the number of people waiting for receiving the second dose, plotted in Figure 4(D), is generally small. These results suggest that the overall preferable solutions, in general, aim at maximizing the number of fully vaccinated individuals by promptly providing the second doses to the population that has already received the first dose and is waiting for it (similar to the trivial alternating strategy proposed in Section 4.1). However, an important exception can be observed. Interestingly, at the beginning of the vaccination campaign, Figure 4(D) shows a period of approximately 3 months in which the optimal solution for the AstraZeneca vaccine tends to inoculate more first doses, thereby postponing second doses. Such a phenomenon (i.e., not observed to the same extent with the Pfizer–BioNTech vaccine), may be due to the good single‐dose efficacy of the AstraZeneca vaccine,^5^ which can be useful in the initial phase of the vaccination campaign to create some sort of (partial) herd immunity, thereby reducing the risk of resurgent epidemic waves.

### 4.3 The effect of the duration of the first dose

Because of the limited amount of clinical data, there is still uncertainty in the scientific community on duration of the partial immunity due to the first dose,^3^, ^5^ and several countries—including Italy—have attempted to delay the second dose.^47^ Hence, we utilize the optimization tool we have developed to explore different scenarios, by changing the value of the parameter α, which captures such a duration. In particular, in view of the observations in Figure 3, we test the AstraZeneca vaccine (see Table 2) by doubling the value of α to α=1/6, and halving it to α=1/24, which represent two extreme cases of short and long duration of partial immunity, which lasts on average 6 and 24 weeks, respectively.

**FIGURE 5:**
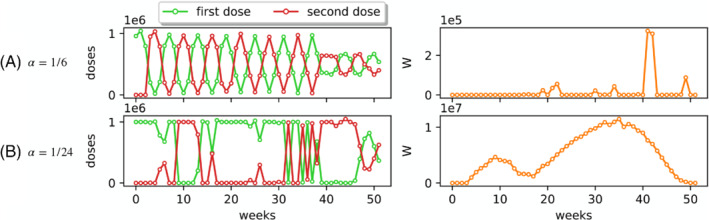
Optimal vaccination rollout for different velocity of loss of partial immunity. The figure illustrates the optimal solutions obtained via the model predictive control for the AstraZeneca vaccine (see Table 2), by assuming (A) a faster loss of immunity that occurs on average in 6 weeks (α=1/6), and (B) a slower loss of immunity that occurs on average in 24 weeks (α=1/24). On the left panels, we illustrates the number of first doses (green) and second doses (red) that should be performed each week, according to the optimal solution. On the right panes, we illustrate the temporal evolution of the number of individuals for which the second dose is being delayed

The optimal vaccine rollouts computed with our optimization tool for the two different values of α are shown in Figure 5. Predictably, the scenario in which the partial immunity due to the first dose has a short duration (α=1/6) produces an alternating solution, in which most of the individuals receive the second doses as soon as possible (W(t) is small), in order to reduce the number of people losing the partial immunity. Interestingly, a longer duration of first dose immunity results in a nontrivial optimal vaccination strategy, characterized by long phases in which first doses are prioritized, thereby delaying the second dose. Such a strategy tends to accumulate individuals in the compartment *W*, which are still susceptible to the disease, but they have a partial immunity, sufficient to keep the number of infections under control and avoid resurgent epidemic waves and the consequential implementation of severe NPIs.

## 5 CONCLUSION

In this article, we introduced a methodological approach to optimally calibrate a two‐dose vaccination policy during an epidemic outbreak. We proposed a flexible mathematical epidemic model that extends the SIR model by adding further compartments to faithfully capture the COVID‐19 epidemic progression and to reproduce a two‐dose vaccination campaign. In particular, we accounted for some key features of the vaccination procedure, including a tunable delay between the first and the second dose and a partial immunity that may be gained after the first dose, but may have a limited temporal duration. Due to the intrinsic nonlinearity of the epidemic process, we propose an optimization framework based on nonlinear MPC, which devises the optimal scheduling of first and second doses for the entire duration of the vaccination campaign. Specifically, the goal of the optimization problem is to design a vaccination rollout that aims at quickly achieving herd immunity while controlling the stress on the healthcare system and allowing the relaxation of NPIs, thereby reducing the socio‐economic costs associated with the pandemic.

We demonstrated our approach by analyzing the 2021 COVID‐19 vaccination campaign in Italy. Our optimization tool allowed us to understand the structure of the optimal scheduling of first and second doses for the two vaccines that are mostly used in Italy: Comirnaty (BNT162b2) by Pfizer–BioNTech and Vaxzevria (ChAdOx1‐S) by AstraZeneca.^46^ Our results suggested that the optimal vaccination rollout indeed entails a nontrivial scheduling of first and second doses, which crucially depends on key characteristics of the vaccine—namely, the efficacy of the first dose to provide partial immunity and its duration—and on the status of the epidemic process. The dangerous cyclical outbreaks (also known as “waves”) that overwhelmed hospitals and the appearance of highly transmissible variants^24^, ^48^ sparked a public and scientific debate on whether the strategy of getting as many first doses as quickly as possible, by delaying the second doses, is medically and strategically sound.^32^, ^49^, ^50^, ^51^ Across countries worldwide, the US and several European countries have committed to delivering the second dose on time for those who received the first dose.^52^, ^53^ A few countries have instead approved guidelines for prolonging the interval between the first and second dose, including the United Kingdom and Canada, which deferred the second dose by up to 12 and 16 weeks, respectively.^54^, ^55^ Several countries—including Italy—are currently pondering this option, as of July 2021.^47^ Motivated by this debate, and in absence of a final word from the clinical community, we leveraged our model to assess the effectiveness of the two strategies, assuming different durations for the immunity induced by the first dose. For the Pfizer–BioNTech vaccine, we found that the strategies in which the delay between the doses is minimized are preferable, and our optimization technique was used to devise the optimal scheduling of the two doses. The results for AstraZeneca showed a different picture, due to reported higher efficiency of first dose immunity.^5^ In this case, as the duration of the immunity induced by the first dose increases, nontrivial strategies with periods of first‐dose prioritization becomes preferable. These strategies are able to create a partial herd immunity that is sufficient to keep the epidemics under control without the need of severe NPIs, while further vaccinations are then performed. In conclusion, our optimization tool suggested that the optimal strategies for the Pfizer–BioNTech entails no delay in the second doses, whereas delaying the second doses of AstraZeneca might be a viable practice, in agreement with the policies adopted by the UK.^54^

When evaluating the outcome of our study, one should carefully acknowledge its limitations. In particular, to keep the model simple and reduce the number of parameters, we made the simplifying assumption that the first dose yields a partial protection against the contagion. However, several studies suggest that, besides this partial immunity, the first dose can also reduce the probability of developing severe symptoms.^56^ While our model can be adapted to this more realistic scenario by adding mode compartments to account for different paths of disease progression, we opted for a simpler model to better focus on the methodological aspects of the study. Furthermore, we assumed that full vaccination is 100% effective in preventing contagion and has no temporal decay. Also in this case, more compartments may be added to account for this phenomenon, at the expenses of the model parsimony. Through the latter addition, our model may be used to support public health authorities to plan an optimal timing for a potential third dose, which seems to be required 9 months after the second, according to some recent studies.^57^ Finally, the model can be extended to incorporate an age‐stratified population,^58^ allowing for the study of prioritization strategies for the vaccination campaign and for a differentiated use of the vaccines, if multiple kinds of them are available. In particular, such a strategy can be devised by extending the control actions $u_{1}(t)$ and $u_{2}(t)$ from scalar to vectors, and adding further compartments to represent the vaccination procedure with the different types of vaccines. To sum up, the flexibility of the proposed model and of the related optimization strategy will allow us to elaborate extensions of the framework without changing its very nature—for instance, by considering uncertainty in the weekly supply—making it suitable to answer a wide range of current and future research questions on the optimal design of vaccination campaigns against COVID‐19 and other airborne diseases with similar characteristics, which may constitute future threats to the mankind.

## CONFLICT OF INTEREST

The authors declare no potential conflict of interests.

## Data Availability

The code and data that support the findings of this study are openly available in GitLab at https://gitlab.com/PoliToComplexSystemLab/a‐model‐predictive‐control‐approach‐to‐optimally‐devise‐a‐two‐shot‐vaccination‐rollout. The nonlinear MPC was implemented using the Pyhton package do‐mpc ^59^ based on CasADi^60^ and IPOPT^61^ optimizer, and double checked using the Model Predictive Control Toolbox in MATLAB.
